# Teaching CAD/CAM/CAE tools with project-based learning in virtual distance education

**DOI:** 10.1007/s10639-021-10826-3

**Published:** 2021-11-25

**Authors:** Rafael R. Sola-Guirado, Guillermo Guerrero-Vacas, Óscar Rodríguez-Alabanda

**Affiliations:** Department of Mechanics, University of Cordoba, 14014 Cordoba, Spain

**Keywords:** CAx, PBL, Applied teaching, Virtualized environment, Design and manufacturing, Skills of engineering students

## Abstract

Computer-aided design, manufacturing and engineering technologies (CAD/CAM/CAE) are a mainstay in today’s industry and therefore they should be an important part in the current training plan of the graduate engineers. However, their implementation in the university environment presents certain barriers that make it difficult. In this work, we study the feasibility of the teaching proposal of the management of this type of tools through a Project-Based Learning method in a distance learning environment. The methodology has been implemented transversally in two Master’s degree subjects related to advanced design and manufacturing and has been carried out thanks to the operation of the product lifecycle management platform software by virtual machines. The practice has given very good pedagogical results in the work of skills related to the field of industrial design and manufacturing. The virtual system has demonstrated high efficiency and students have shown a satisfactory evolution in their professional training.

## Introduction and background

In recent years, Computer-aided Manufacturing (CAM) technologies have definitively been integrated as developing and evolving body within the manufacturing industry. Now the products are designed and calculated virtually by means of Computer-aided Design and Engineering (CAD/CAE) techniques, simulating the conditions to which they will be destined. With the adequate use of these software platforms, product development is achieved with minimum costs and a rigorous fulfilment of the required functionalities along with the most optimal manufacturing process (Li et al., 2020). The phases of the complete life cycle of a product are supported by various information technology systems. Major advances in the field of computation, new trends in the industrial marketplace and the evolution of the information and communication technologies (Wang, 2018) forces to train the new design engineers in an ecosystem according to the system where they will perform their work (Morales-Avalos & Heredia-Escorza, 2019), and these industrial software platforms are suitable for this. Platforms tend to shift their way of working to the cloud, with different highly specialised modules, allowing multidisciplinary and collaborative design work. This new paradigm requires studying the feasibility of this operability for academic purposes. The COVID-19 pandemic has led to the adaptation of traditional teaching models, as systems with individual licences in the classroom are no longer possible and teaching approaches with more global design techniques are being addressed.

### Use of CAx in the actual context in higher education

The use of CAx (Computer-aided technologies) in higher education, complemented with virtual environments, has led to the beginning of the training of technical design specialists at the upper-secondary schools (Kuna et al., 2018) and these systems must inevitably be implemented in higher education. However, the implementation of these software platforms in the universities involves certain technical and economic difficulties, requiring extra time to simultaneously teach their function and the specific practical knowledge. Today, many academic institutions offer single-user licenses that lead to certain problems such as the need for specific hardware and software. In addition to these difficulties, there are also barriers and inconveniences caused by the fact of working outside the classroom. Conversely, in today’s industry there is a trend towards cloud-based distributed systems due to the evolution of intranet systems and software and the great advantages in terms of ubiquity and security that they offer (Wu et al., 2015).

The COVID-19 pandemic has markedly affected the education at high levels. At the beginning of 2020 the situation forced the universities to quickly adapt their teaching methods. Thus, students and teachers have had to prepare for this forced transition from classroom teaching to remote teaching online. The need to make new learning environments available in the current learning system and the design of new media adapted to teaching were the main challenges for the University centres and for the teachers, respectively (Carrillo & Flores, 2020). In the case of the University of Cordoba accommodated teaching to the new situation, designing a protocol for teaching action and investing in the installation of systems to be able to teach master classes in synchronous videoconference mode. A new Virtual Desktop Infrastructure (VDI) system for the use of CAx software platform applications, among others, has been implemented and will be studied in this work. The configuration of the software platforms available to students and teachers on the virtual campus and the competencies of the faculty such as their digital skills have been key factors in maintaining good teaching performance (König et al., 2020). This pandemic situation has accelerated the changes that were expected to occur in the coming years and has shown that learning virtual is has given the possibility to evaluate e-learning with these new virtual tools.

### Training new engineers using CAx in an active learning environment

The use of these new platforms also means that the teaching models used must be adapted. This condition has made a new planning of practical activities essential in order to optimize student learning and adapted to the new system. In order to adequately train new professionals in this field, different models are required from the traditional ones based on reverse engineering, which deal exclusively with product modelling (Sola-Guirado et al., 2020). These models spend a lot of time on the operation of the software’s own commands, limiting the performance of the learning acquired by the student (Otto & Mandorli, 2018). Thus, useful time is subtracted for the assimilation of other competencies more involved with reverse engineering, i.e., using technical data to design a certain new product, or optimizing it according to certain requirements (Daly et al., 2012). Learning should not be limited to the development of general geometric modelling and calculation skills, but to a broader spectrum of competences focused on knowing how to manage all available information to create functional products, thus promoting the creativity of students and improving their training in skills and competencies in the field of product design and manufacturing.

CAD expert learning requires a shift away from behaviourist methods, as an orientation towards command knowledge is detrimental to the learning of other strategic knowledge (Chester, 2007). This hypothesis has already been supported years earlier by Lang et al. (1991) when they argue that students spend more time learning commands than acquiring other types of information such as procedural knolwledge. The results of traditional teaching in academia translate into engineering graduates who are perceived by industry as incapable of solving problems due to the lack of their practical approach (Dutson et al., 1997). This suggests that engineering designers need skills that help them deal with complexity. In this sense, Dym et al. (2005) propose working on thinking about the beahiour of the system dynamic, reasoning about uncertainty, making estimates and conducting experiments. All this is based on ‘Design Thinking’ as a series of continuous transformations from the concept to the knowledge domain.

Leading universities have demonstrated the benefits for this purpose with the use of techniques based on ‘Active Learning’ (Hernandez-de-Menendez & Morales-Menendez, 2019). In this sense, the active learning methodology involves addressing problems and developing cognitive abilities, increasing motivation and fostering student curiosity and creativity. Works consulted demonstrated that cognitive learning is directly related to the assessment of learning tasks by students. This approach is closer to the actual product development process and works on skills required in engineers such as knowing how to search for information, synthesize conceptual designs, combine aesthetics and functionality, etc. Furthermore, it adapts well to the changes demanded of design engineers towards converging disciplines and supports collaborative work (Abdullatif Almulla, 2020).

Project-based Learning (PBL) has proven to be a very valuable strategy for developing the competencies addressed in learning product development subjects in real work experience (Barbero & García, 2011; Berselli et al., 2020; Pindado et al., 2018). Students can develop a certain product in the scope of a project using the usual tools of the industry in a learning environment. At the same time, the individual’s developmental capacity to solve a common problem statement can be enhanced (Stolk & Harari, 2014). On the other hand, when students need to solve complex open-ended design problems, teachers have less time to dedicate to each student to solve their specific problem, and they are the ones who have to deal with them the most (Xie et al., 2018). However, design is an inventive process and therefore, it should be worked on as such, with activities that require this skill (Cropley, 2016) and work on skills based on visual mental imagery. Recent studies in virtual environments encourage the use of PBL techniques to improve students’ competences and motivation (Aslan & Duruhan, 2021; López et al., 2020).

According to the approach described above, new engineers need adequate training in CAD/CAM/CAE tools according to the needs demanded in the industry for the development of new products. The trend in the work of this type of engineers is towards collaborative work with software in the cloud. Therefore, their university training in this field with virtual environments combining active teaching strategies can be a suitable approach to improve their competences.

The present work proposes a novel framework based on PBL consisting in the realization of one project in design and manufacturing matters that starts from some technical premises with which the students will have to get involved in a real process of product development. Specifically, this project is intended as a case study carried out in different master subjects using the CAD/CAM/CAE tools by a virtualised environment and the work outside the classroom, due to the COVID-19 situation. The objective of this work is to evaluate the usefulness of this work strategy to obtain a high performance in postgraduate teaching using CAD/CAD/CAE tools through a practical methodology and distance work so that students can learn by doing and enhance the assimilation of global competencies. Likewise, this work describes the basis and functionality of the cloud-based server system and aims to discuss its impact on distance learning.

## Methodology

The present work has been planned and developed on the basis of the definition of a traditional versus a PBL-based approach in product development matters; the description of the infrastructure implemented for the application of the new PBL-based programme of activities using distance learning; and the evaluation of the method and technological resource for this joint purpose.

**Fig. 1:**
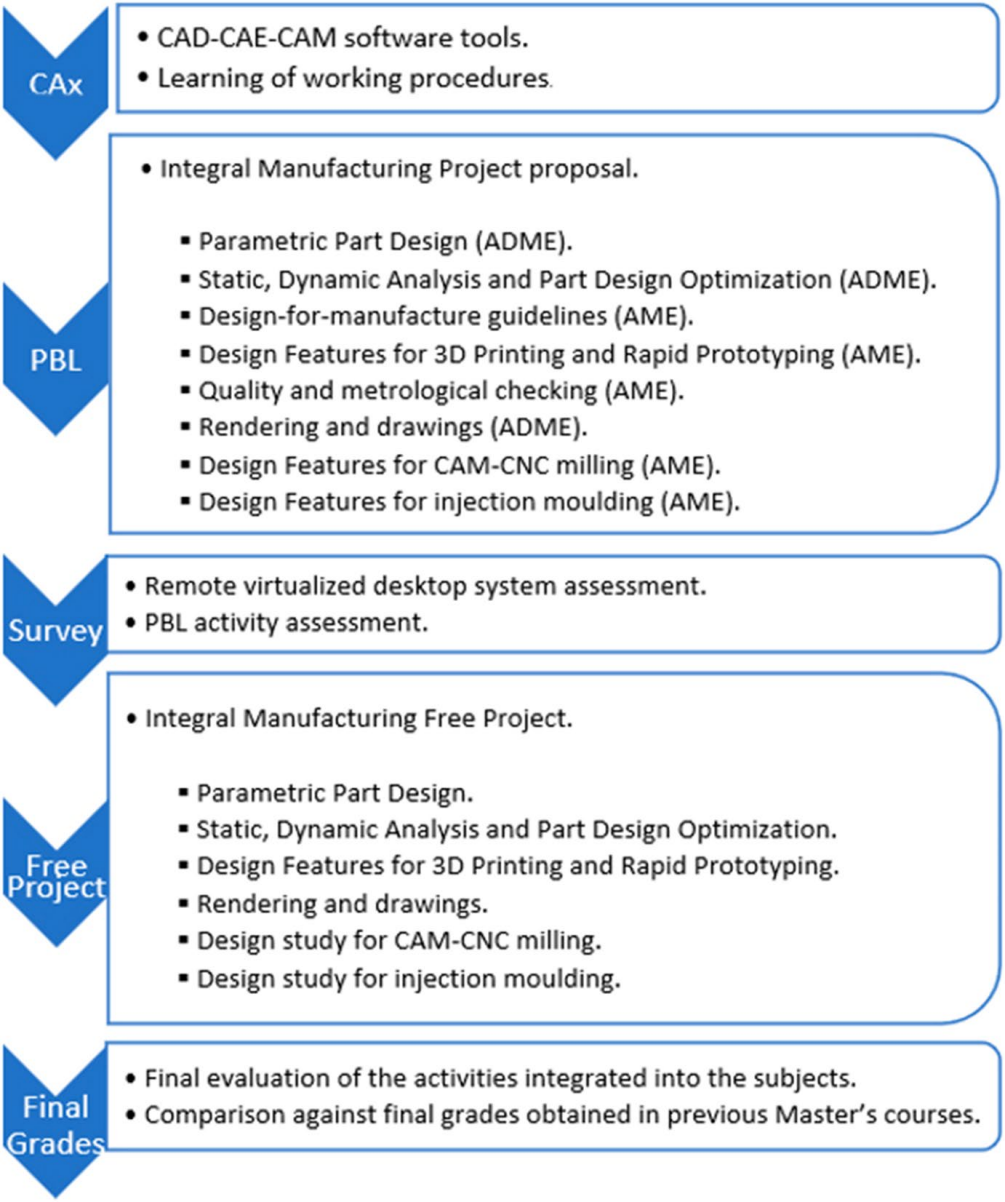
A summary outlining the steps followed during the Master’s course

In a first stage of the course, the students received detailed information on the CAx tools and working procedures to be used (presentation sessions). In a second stage, a common manufacturing project “robotic-mechanical gripper” has been proposed. This common project, divided according to a series of competency substages, has been developed according to a coordinated session planning in both subjects (shown in Table 1). The remote virtualized desktop system, used in all the sessions, and the PBL proposed activity were assessed through a specifically designed survey aimed at students. This evaluation survey was completed at the end of the execution of the proposed project. In this way, all the students were informed that the results they obtained would be published blindly and that they could exercise their right not to participate in the present study. No objection was received. Finally, the teaching team involved in this work was able to verify a notable improvement in the results after evaluating the final free-choice projects developed by each student in the context of these subjects. In this sense, a comparative study of the students’ final grades obtained when working by the traditional procedure (2018 and 2019 courses) versus final grades obtained by the PBL approach (2020) corroborated this improvement The Fig. 1 summarizes the learning process.

**Table 1:** Sessions and activities proposed with the traditional approach shown by subjects

Subject	Week	Learning module	Description of teaching
ADME	1	Industrial design and CAD	Basic industrial design theory and techniques and its application by CAD
AME	1	Industrial manufacturing and CAM	Basic manufacturing theory and its application by CAM
ADME	2	Solidworks enviroment and 2D drawings	Toolbars, menus, comands and operations to create 2D elements
AME	2	Solid modelling	Different operations to create/modify 3D solid elements
ADME	3	Solid modelling	Different operations to create 3D solid elements
AME	3	Topological design & optimization	Design a part for a good functionality, material saving and efficient manufacturing
ADME	4	Rendering and drawings	Obtaining photorealistic images and manufacturing drawings from an assembly
AME	4	Prototyping using 3D printing	Preparation of one part for 3D printing
ADME	5	Static and dynamic system analysis	Simulations of parts under dynamic stresses and loads
AME	5	Quality and metrological checking	Geometric, dimensional, and surface quality controls
ADME	6	Assembly and kinematic simulation	Mechanical constraints between parts and obtaining motion simulations.
AME	6	CAM with milling or molding	CNC programming and process sheets

### Context of the implementation of the work

The approach proposed was set in two subjects of the Master of Industrial Engineering of the University of Cordoba: Advanced Design in Mechanical Engineering (ADME) and Advanced Manufacturing Engineering (AME). This master’s degree enables students to develop the skills required to obtain the title of Industrial Engineer. The master’s degree is divided into four modules: industrial technologies, management, installations, plants and constructions, and final master’s thesis. The master’s degree is awarded a high level of academic training, MECES level 3 in the European Higher Education Area.

Each subject had a duration of 3 ECTS credits. In these subjects, CAx technologies are taught as tools for virtual design and manufacturing using software such as Autocad, Solidworks, Ansys, MasterCam, etc. and the training of the students in their lasted about 6 weeks with 2.5 h per week. Each academic year and each subject was taken by groups of about 25 common students in both subjects.

The present work has been focused on the assessment of the results obtained by the application of a traditional teaching approach in computer classrooms at the University with a local connection carried out during 2018 and 2019 versus the new approach using the PBL methodology in a virtualised software environment used during 2020.

According to the syllabus, the main competences that are specific to the work of a Product Developer and that students should get by both subjects (ADME and AME) are:

- Knowledge of engineering software.
- Knowledge of TICs and reseach skills.
- Calculate and design products and processes.
- Make judgments based on information.
- Problem solving within broader contexts.
- Knowledge of the scientific and technological aspects.
- Machine design and testing capabilities.
- Evaluate the effectiveness and quality of solutions.
- Plan the effective development of projects.
- Project, calculate and design products and processes.
- Communicate findings and reason them.

However, with a traditional approach that spends a lot of time on software based on learning commands, it is very difficult to work on a large number of them, but with a PBL it is expected to work on them in a more specific way. Therefore, in this paper, an evaluation of the development of these competences with both teaching approaches will be carried out, as detailed below.

### Traditional teaching vs new approach (PBL in VDI)

**Fig. 2:**
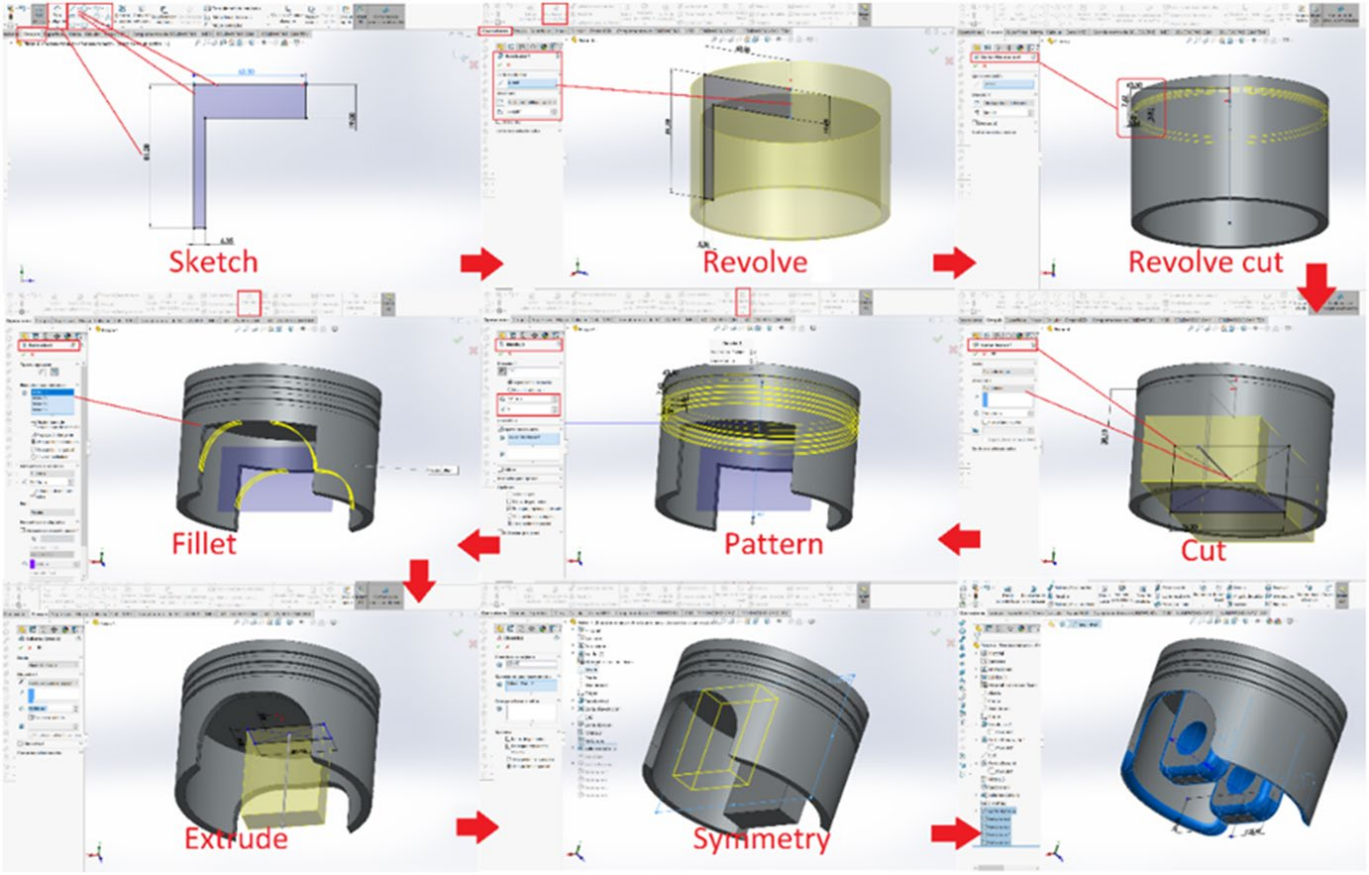
An isolated (3D modelling) practice developed within the traditional approach

The traditional approach consisted of theoretical lectures explained the software command complemented by a program of transversal practices consisting of several exercises with learning guides practices. These learning exercises and the mechanical components addressed in them were not connected and unrelated to each other, and were solved in class in a step-by-step guided manner. During the master classes, the teacher guided the student sequentially through the steps they had to take to advance in the resolution of each exercise, commenting on the commands to be used and solving all the doubts and questions raised during the class. In this way, the final result obtained by all the students should be the same, if they had carried out the process correctly. The Table 1 shows a summary of the sequence of sessions and practice activities developed during the courses 2018 and 2019, following the basis of the traditional approach. In the Fig. 2 an example of a typical exercise in the design of an isolated mechanical component is shown. This methodology aims to teach the student the fundamentals of applied manufacturing techniques as well as the operation and management of different software tools in the field of design and manufacturing, but using engineering problems which do not have an interrelation or are not related. Integrated into a common project.

**Fig. 3:**
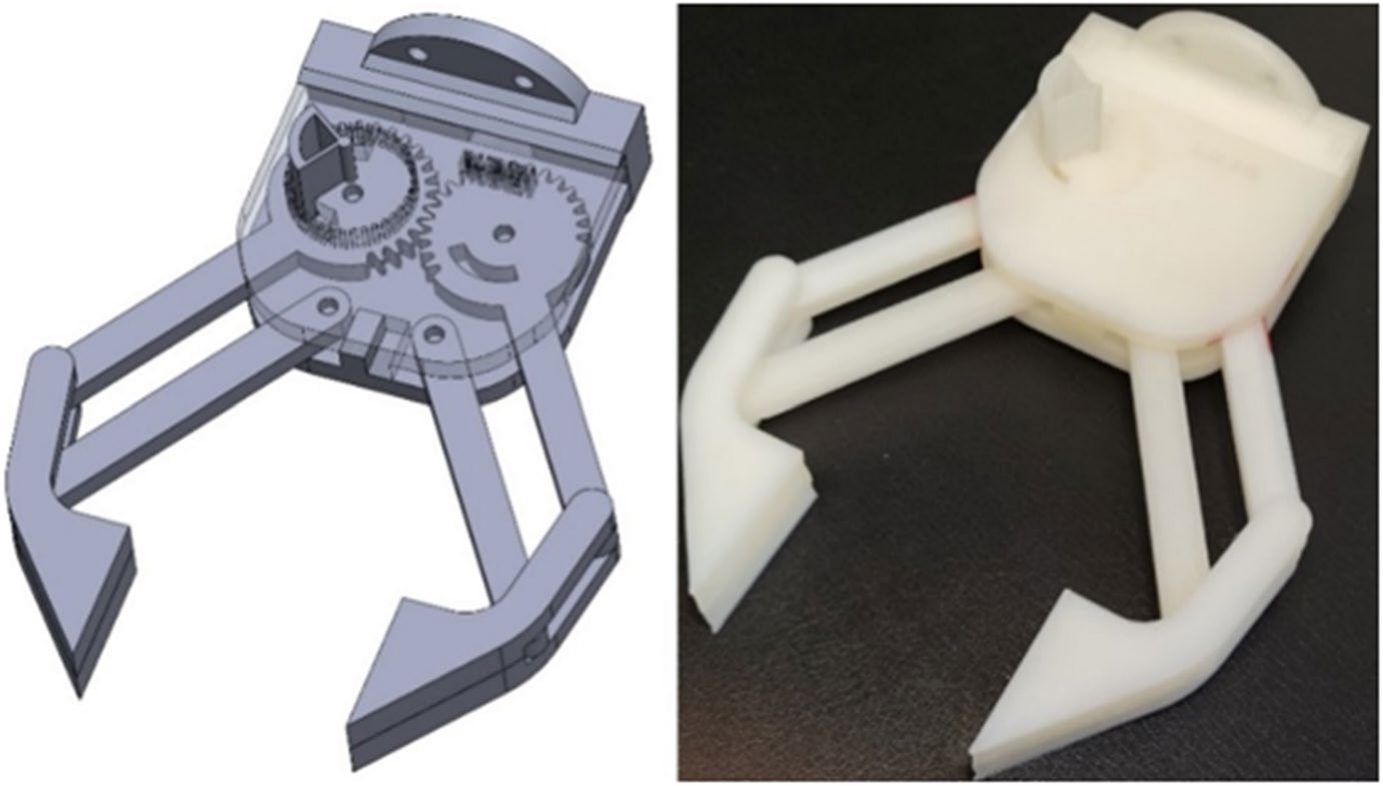
Virtual model and prototype (gripper) used by the teacher for the PBL

**Fig. 4:**
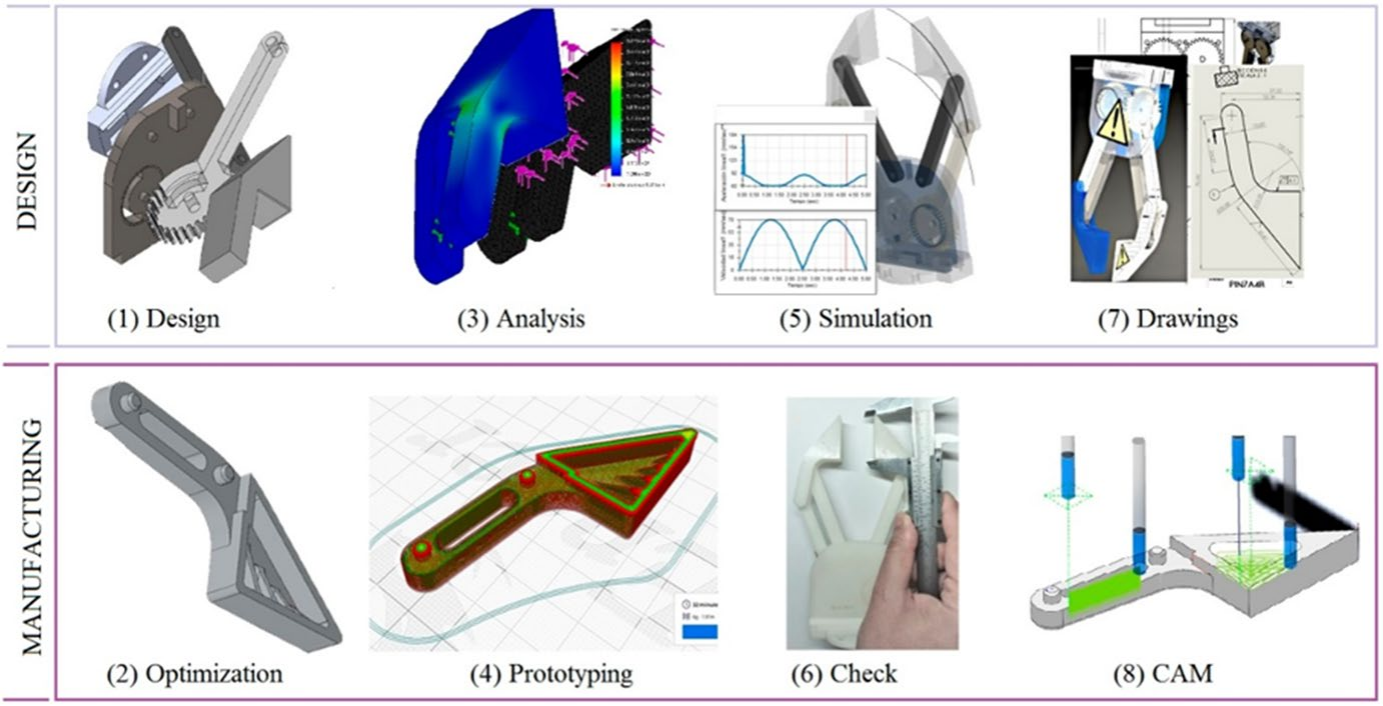
Different phases proposed in PBL (according to the sequence shown in Table 2)

The new teaching methodology changed their traditional teaching model towards the PBL approach that addresses the realization of a common project between the two subjects in an organized way. In particular, it has been proposed to develop an arm gripper to be inserted in a robotic arm that allows the gripping of a part. A design model proposed by teacher is shown in Fig. 3, from which students can orient themselves in order to be able to realize their own designs. The teacher approaches the first 30 min in class by making a quick simulation of the tasks to be done, exposing an example of the final result. Figure 4 shows an example of the partial results obtained that students should achieve by learning modules within the PBL. For that, the project was sequenced in different stages that force the students to go through the product development phases (Table 2). Afterwards, the students must individually do their projects with their own specifications, working in sessions based on synchronous face-to-face classes through online videoconference where teacher can help them in all the product development process.

**Table 2:** Sessions and activities proposed in the PBL distributed among the subjects. * In brackets are indicated PBL sessions linked with Figure 4

Subject	*Week	Learning module	Description of teaching
Common	1	Fudamentals of Solidworks	Environment, modules, design and calculation and specific commands
Common	2	Research and market study	Search tools for technical and market information
ADME	3 (1)	Parametric design of machine parts	3D CAD design of parts of the gripper with different solid operations
AME	3 (2)	Topological design & optimization	Modify the gripper parts for a good functionality, material saving and efficient manufacturing
ADME	4 (3)	Static and dynamic system analysis	Simulations of the gripper parts under dynamic stresses and loads that will occurs
AME	4 (4)	Prototyping using 3D printing	Preparation of the gripper parts for 3D printing
ADME	5 (5)	Assembly and kinematic simulation	Mechanical constraints between gripper parts and obtaining motion simulations. Physical assembly of printed parts
AME	5 (6)	Quality and metrological checking	Geometric, dimensional, and surface quality measurements from the printed parts
ADME	6 (7)	Rendering and drawings	Obtaining photorealistic images and manufacturing plans for the real gipper
AME	6 (8)	CAM with milling or molding	Obtaining programs and process sheets for manufacturing using CAM

All this was done remotely through sessions with Cisco Webex Meeting ® platform offered by the university, where the contents are shared effectively, annotations can be done on the screen and even the control of the student’s virtual machine can be taken if needed. The students are connected by their personal computer with an own internet connection and through the Virtual Desktop Infrastructure (VDI) platform outlined in the following section. Thus, they can use their personal credentials to get access to the SolidWorks CAD/CAM/CAE software platform, which offers an environment very close to those of Product Lifecycle Management (PLM). The modules that the students worked on in the development of the project were:

- CAD: Solidworks 3D Part Modelling, Assembly, Drawing and Toolbox.
- CAE: Solidworks Simulation: static and fatigue analysis, modal and topologic study.
- CAM: Cura 3D Printing: programming, simulation, process planning and documentation.
- CAD/CAE: SolidWorks Motion: mechanism analysis.
- CAE/CAM: Solidworks CAM and Solidworks Plastics: process programming, simulation, planning and documentation.

#### Evaluation of the students performance in the subjects

The evaluation of both subjects corresponding to the exposed CAx modules (not addressed in this paper) was carried out in two ways. On the one hand, a 50% evaluation of the practical exercises guided with the traditional approach or of the proposed project with the PBL approach. On the other hand, a 50% evaluation of an individual project of each student (with a point of differentiation with respect to those similar ones that exist on the market) in which they must Design (ADME) and prepare some parts for Manufacturing (AME), going through the phases and in a coordinated way between both subject. For the latter project, students have 2 weeks of work at home and it has to be presented in a short “Pecha-Kucha” format at the end of the course. Finally, the student obtains a final official grade of 0 to 10 points in the subject.

#### Infrastructure for the development of the PBL teaching plan

**Fig. 5:**
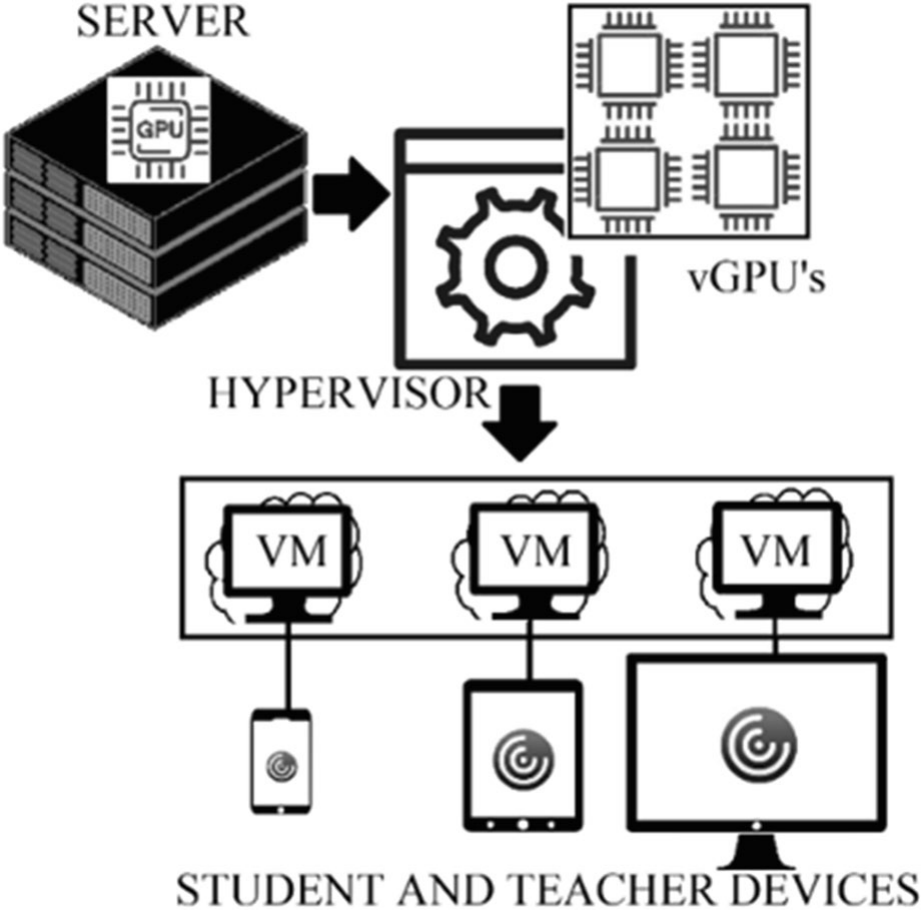
Representation of the platform system used for remote virtual learning

A virtual desktop infrastructure generated by Citrix machine creation system has been configured. The system presents users with remote desktop sessions through a Citrix XenDesktop platform and Citrix Workspace client (Citrix Systems, Inc., United States) running on virtual machines (VM) through a VCenter virtualization platform (VMware Spain, S.L., Spain). For this, servers HPe Synergy 480 Gen10 (Hewlett Packard Enterprise, Spain) are used to run VSphere (VMware Spain, S.L., Spain) which are equipped with graphics cards nVidia Tesla p6 (NVidia Corp, USA). The representation of the graphics is done in the virtual machine itself, and therefore, in the host server that hosts them sharing the hardware resources, while the presentation is done in the user’s computer. The virtual graphics processing unit (vGPU) used, virtualizes and shares the allocation of the host server’s physical GPU among all the VMs, as well as the rest of the resources, including the central processing unit (CPU) and memory. The vGPU are reserved and released as the VMs are turned on or off by each remote user. The hypervisor system is responsible for managing the active VMs through the different open hosts and in such a way that these are not blocked. For this, the VMware management and control platform uses a distributed resource planning system. All of this, allows users with university credentials to connect to the platform from any device with Internet access (Figure 5).

The machine creation system allows the design and delivery of VDI images by copying a master VM and generating linked clones to provision multiple virtual desktops. The disk of each of these VM clones stores only the differences from the master shared VM disk information optimizing both disk space and cloning time. The VMs for the remote desktop have been sized with 8 Gb of memory and 4 virtual CPUs, assigning a 150 Gb disk for each of them. The host servers were assembled in a chassis forming a ‘blade’ style ‘composable’ architecture, where the different servers share power, network, storage, administration and other resources. The installation consists of 6 hosts and each one has 2 CPUs with almost 4 TB of memory. The hosts have several 10 Gb interfaces that are concentrated in a virtual switch in the chassis itself, which is redirected to the general core of the network through two 40 Gb interfaces. Internal communications between them remain within the virtual switch itself. The hosts mount their storage in an unity disk cabin Dell EMC (Dell, USA) equipped with solid state disks. This cabin exports the different DataStores to the virtualization infrastructure, as well as the network shares used for tasks such as Windows and Linux user homes, profiles, etc. The connection of the hosts to the store cabin is done through eight fiber channel interfaces at 8 Gb of bandwidth each. This entire infrastructure, managed through Citrix’s centralized interface, also makes it possible to monitor usage, identify possible errors, provide user assistance, extract statistics, etc.

The last component of the system is the licensing of non-free applications and their corresponding floating license servers that allow them to be shared between the different sessions, saving costs as opposed to having them assigned to physical PC units. In this case, SolidWorks software (Dassault Systemes, France), which has been mainly used in the proposed learning plan, offers diverse CAx software modules, integrated into a single platform that enables to develop all the project stages in a common environment, thus facilitating the student learning. With this software, the presentation of the virtual desktop is compatible with the H264 compression techniques, something that is essential in the handling of this type of software aplication modules. Depending on the speed of the network from which the client is connected, these compression systems will be more or less effective.

### Assessment of teaching method and the virtual platform

An evaluation of the teaching methodology and procedure applied through the implementation of the PBL has been approached through a survey (Table 3) designed specifically to determine the practical and pedagogical potential, advantages and drawbacks for use in computer-aided design and manufacturing software applications integrated in the virtual enviroment. On the other hand, the virtualized desktop system has been evaluated too. The survey have been made available to the students at the end of the course using the Google Forms tool and has been carried out and treated confidentially. The weighting in each of the questions has been established between 1 (very low) to 5 (very high). Besides, it has been possible to assess the proposed practical activities, determining its applicability in a distance environment within the platform used, obtaining a useful feedback for the integration of transversal activities between related subjects. The results will serve as feedback to optimize the implementation of all resources, as well as to improve the accessibility and performance of the software applications used. The withdrawn experience will allow to study its usefulness and effectiveness in order to improve the students’ learning, motivation and creativity.

**Table 3:** The blocks and objective questions that make up the survey implemented

N.	Blocks and Objective questions
Teaching by PBL methodology	
1	Degree of motivation reached with the use of the project.
2	Usefulness of the methodology for the assimilation of the skills designated in the teaching guide.
3	Degree of understanding of the theoretical and practical knowledge.
4	Utility of the activities to manage efficiently the CAD/CAM/CAE tools.
5	Coordination and transversal interrelation between the subjects.
6	Difficulty of learning due to distance education.
Virtual platform for the teaching and virtual CAD/CAM/CAE tools	
7	Adaptability and ease of use of the virtualized environment.
8	Functionality of platform applications from different locations and with different types of devices.
9	Agility and computational performance of specific applications and functions.
10	General assessment of the Solidworks software modules and applications and their functionality.
11	Assesment of the remotelly licensed software use vs. unlicensed or individually licensed software.
12	Interest in having other engineering software integrated into the server.

In addition, a evaluarion has been carried out by the teachers in order to evaluate the skills or competences obtained by the students through the application of the traditional approach and the PBL approach with virtual distance. For this purpose, the results of the final projects (section 4.2.1) that were developed both in the course with traditional teaching and in the course carried out with the proposed new approach were evaluated.

## Results and discussion

**Fig. 6:**
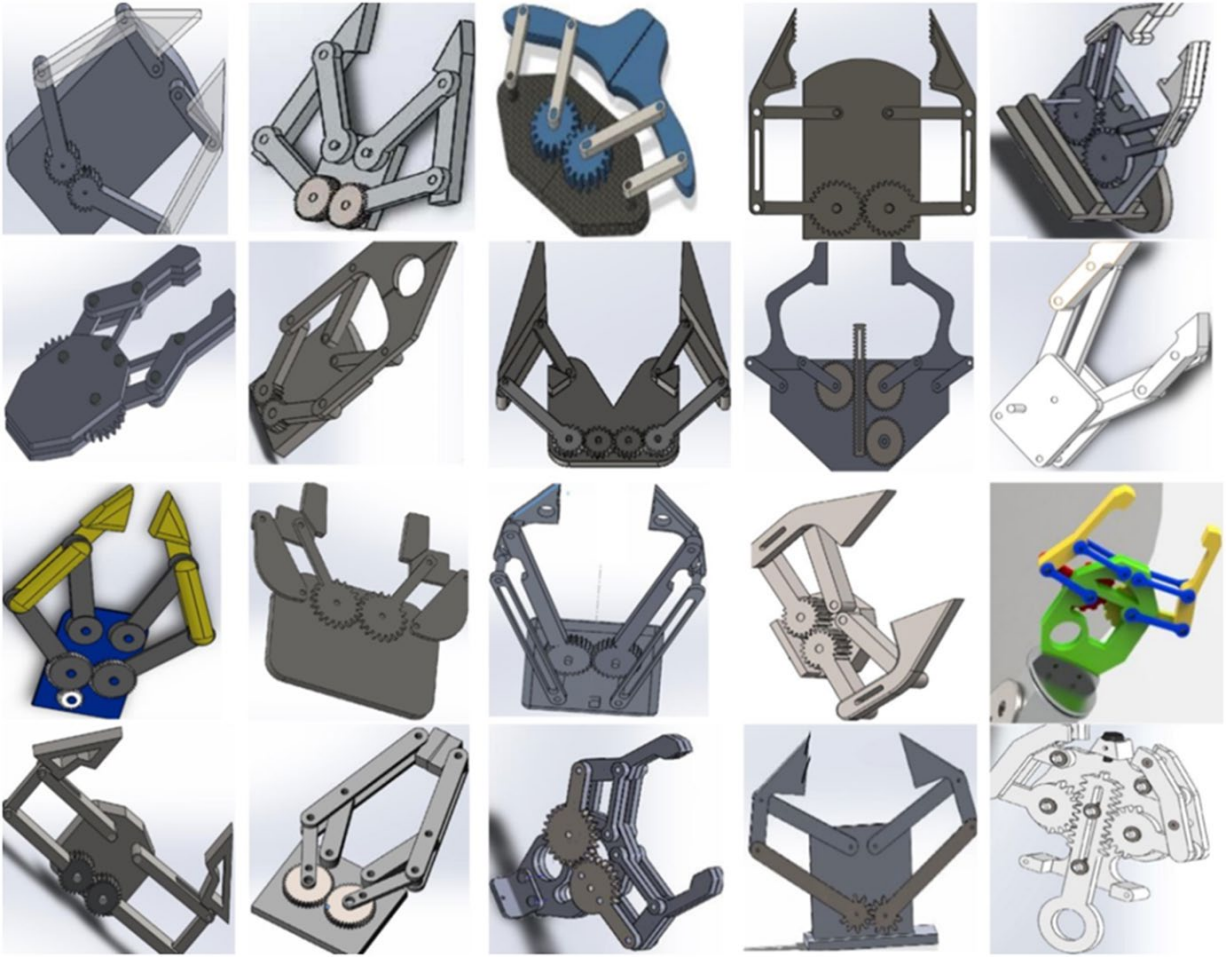
Examples of results achieved by the students in the different sections of the PBL

The approach shown by the teacher throughout the different work sessions sequenced as shown in Table 2 and Fig. 4 has been very useful to clarify to the students what they should work on and how they should do it. The students made, in general, some prototypes highly suitable to the objectives pursued (Figure 6). The project presented in this work overlap most of design philosophies, listed by Ye et al. (2004): ‘design philosophy’ addressing bottom–up design, ‘parametric modelling’ to create product models by means of its features ‘feature-based modelling’, ‘concurrent engineering’ integrating design and manufacturing, ‘network-centric design’ by using the current technologies and ‘creative design’ leaving the final solutions open to the student’s own ingenuity. Many institutions deal with the different phases of design in their teaching (Lorenzo-Yustos et al., 2010) but few integrate them into a common project such as this one, which shows an integral perspective of design.

**Fig. 7:**
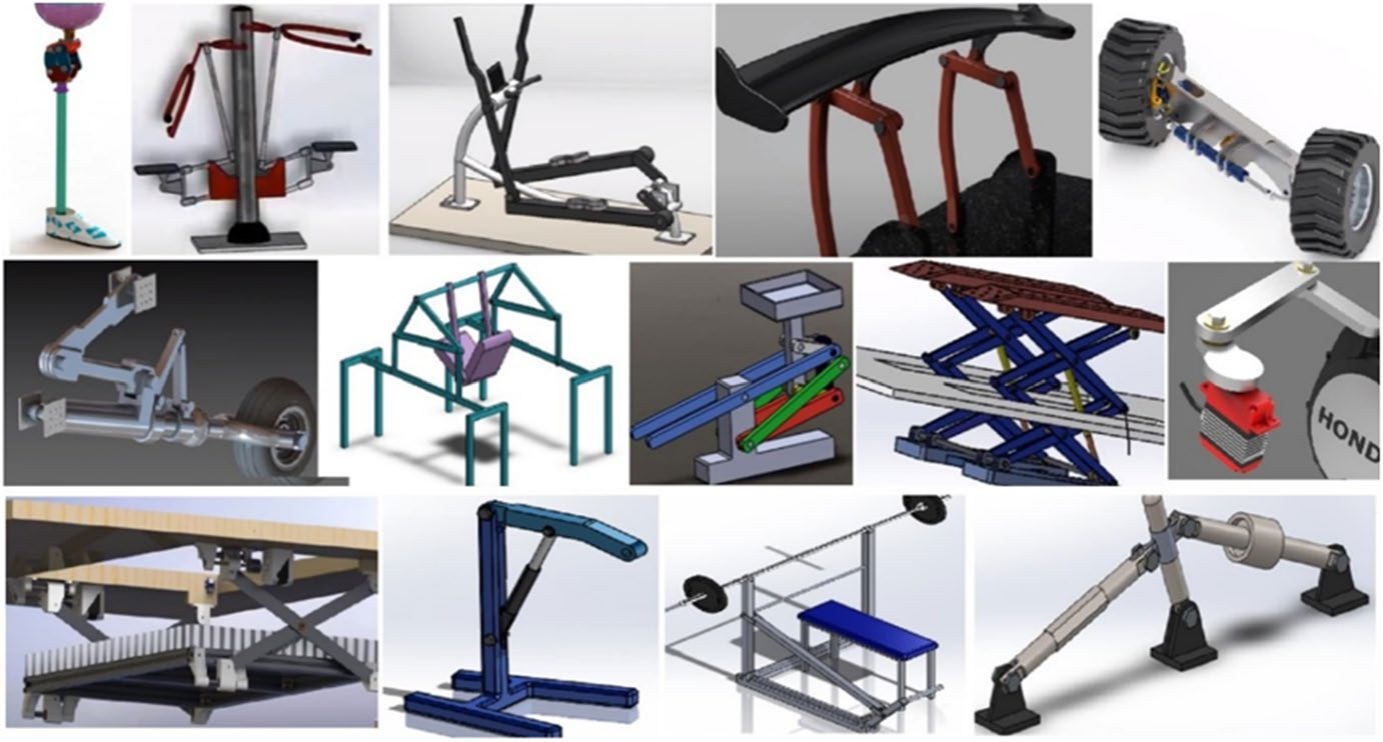
Examples of results achieved by the students in their final projects after using the PBL approach

As a significative result, students completed their final projects with a high degree of efficiency and, according to personal interviews conducted at the end of each course, with a higher degree of ease than in courses with traditional teaching. Some examples of these student individual projects are shown in Figure 7. In fact, students’ final grades, on base 10, improved from 6.1 ± 1.6 to a 7.6 ± 2.3 (Mean ± SD) in the case of ADME and from a mean 6.8 ± 1.5 to a 7.7 ± 2.6 in the case of AME, when moving from the traditional procedure to the PBL approach.

**Fig. 8:**
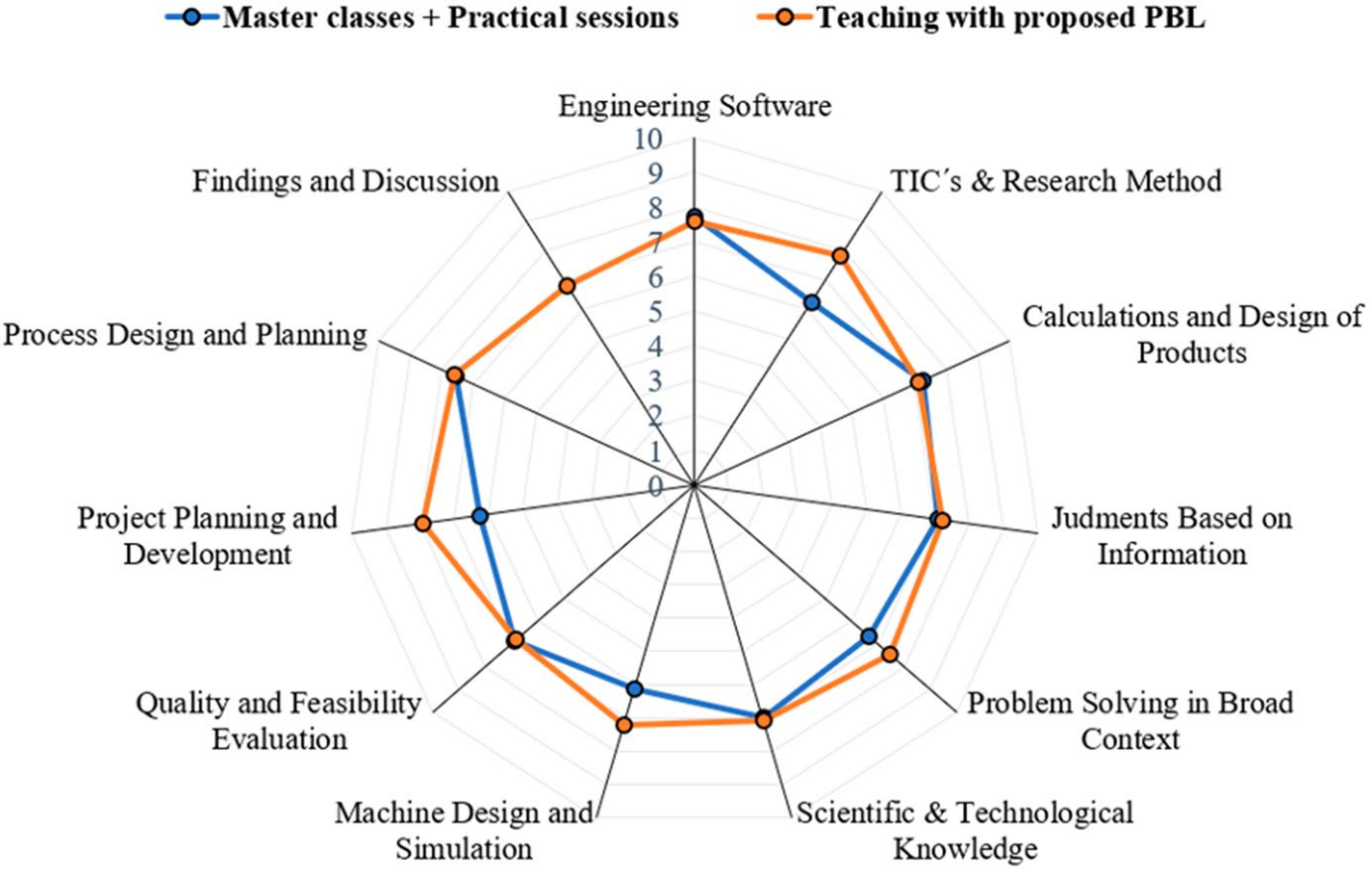
Skills and competences acquired by the traditional method and by the implemented PBL methodology

Figure 8 shows the performance results obtained in certain competencies (Table 1) that are objective in the different subjects, after a teaching with traditional approach and with the approach proposed in this work. The results show the effectiveness of the new methodology used for learning, improving on the performance of previous years, increasing overall from a 6.86 ± 1.2 to a 7.33 ± 1.06 (mean ± SD). The actions carried out in this activity promote learning according to Merrill (2009) as learning occurs when students are engaged in solving real-world problems, activated as a basis for new knowledge, demonstrated by someone like the teacher and experienced by themselves. The PBL allowed students to acquire the competencies provided in the teaching guides, helping to enhance other skills such as creativity, research, solving broad problems, design skills understood as creation and planning skills. Evidently, the PBL methodology is not focused on issues such as specific knowledge of the software applications or knowledge about the implicit calculation processes.

**Fig. 9:**
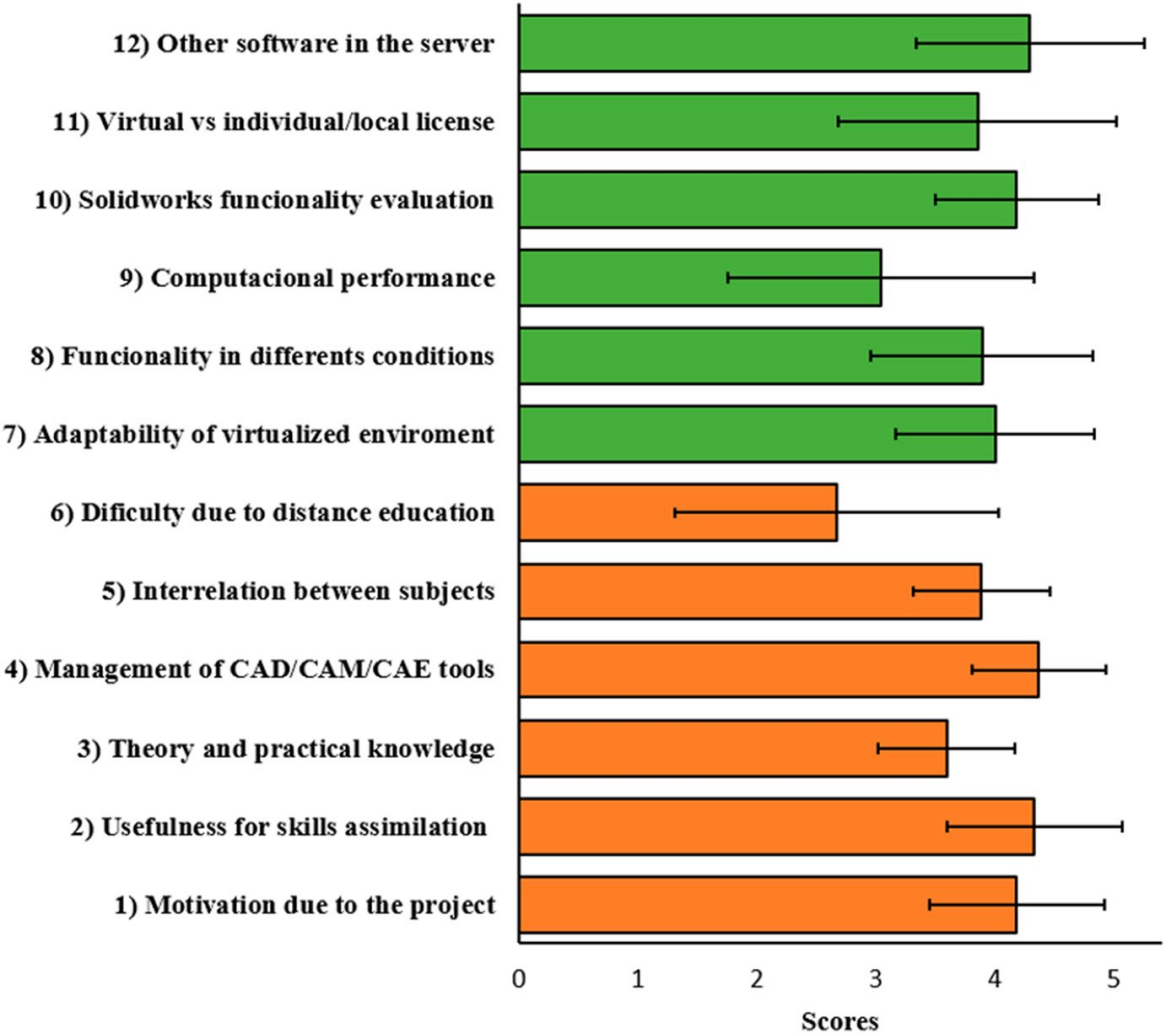
Scores obtained (mean values + standard deviation) from the survey for the evaluation of the implemented on PBL methodology (questions 1–6) and the use of the virtual platform employed to work with CAD/CAM/CAE tools (questions 7–12)

Finally, the students have positively evaluated both the methodology and the virtualization system as shown in the results obtained from the survey designed for this purpose, as is summarized in Figures 8 and 9. The results indicate a high level of student satisfaction and a very high improvement of their motivation in subjects, scoring 4.19/5.

This is in line with other studies that point out the good perception and assimilation of students with PBL strategies in engineering learning (Belagra & Draoui, 2018; Ricaurte & Viloria, 2020). This type of project is very beneficial for fostering creativity (Wu & Wu, 2020), competence that is commonly neglected in engineering education. The students recognize that the most motivating part of the project is being able to make their own decisions in their designs (Foley & Kazerounian, 2008). In fact, although the designs made by the students (Fig. 5) have great similarities due to its inherent limitations and the conditions imposed in the exercise, there are notable differences due to the different personal creativity and interpretation of the same design problem by of each student.

The methodology used has been highly useful for the assimilation of the competences set out in the course, obtaining a score of 4.33/5. On the other hand, it seems that the general knowledge of the theoretical contents, although good, is not so high (3.59/5). It must be said that the learning method used by the teacher dictates the assessment to be used, so it is different to use a theoretical or practical approach, as presented in this work. In today’s society, engineers are faced with a variety of different problems, so the use of a single learning method based on a single educational theory is not the most appropriate (Hassan, 2011). Perrenet et al. (2000) pointed out PBL as a powerful tool to create a link between theory and practice in a gradual way, but there are no decisive studies to weigh in what proportion the theoretical and practical classes should be complemented for better learning (Hamade et al., 2009). Thus, the present study focuses on its evaluation to get to know the adequate balance between the implementation of traditional learning method and PBL based method.

The proposed project deals with a large number of phases in the development of a product and the interrelation with the two related subjects (design and manufacturing), valued at 3.89/5, enables the student to tackle the same problem from global perspective and closer to the real industrial environment. This relationship between subjects must be approached with special care, as it is the main limitation for correctly implementing a PBL strategy (Perrenet et al., 2000). The adequate sequencing of the different activities carried out has allowed the students to handle a large number of CAD/CAM/CAE software tools. Although the time available in the subjects is not very long for a fluent handling of these tools, the students rate this section very positively with 4.37/5. As far as the evaluation of the Solidworks software platform itself is concerned, the students gave the software a very high score (4.19/5). The students have been able to value the potential and functionality of the software platform and, in their future professional life, they can improve they will be able to improve their skills based on work and experience.

Although the experience has been enriching, it seems that students do not have a clear predisposition between distance or face-to-face education, since they score this aspect in relation to 2.67/5. This reflects the change in the paradigm of engineering education where the teacher is no longer a transmitter of information but a mentor, coordinator, and facilitator of learning (Hardy & Bower, 2004). This forces a change in the traditional role of teaching based on text-reading and teacher-centred learning (Boling et al., 2012), and therefore, strategies such as those presented in this work are perfectly suited to distance learning. Osei and Mensah (2011) pointed out that distance learning in engineering courses does not detract from student performance. However, in order to get a quality distance teaching that allows to improve this neutral opinion by students (face-to-face vs. virtual), it is essential to have a good computer and network connection systems.

The virtualized environment developed seems to have a good adaptability and a user-friendly interface and handling, scoring 4.00/5. The virtual desktop infrastructure (VDI), since it does not require a fixed terminal, has allowed its remote use. The operation of VDI, which works under Windows OS, means that all applications developed for this operating system can be executed on this type of server, while on a Remote Desktop Service terminal server not all conventional applications can be executed (Mousa, 2012). Desktop virtualization technology provides a great flexibility in educational organizations, with a number of associated benefits such as ubiquity, accessibility and optimization of computing resources. The implemented platform has allowed students and teachers to connect remotely through their devices in an efficient way, scoring 3.89/5 in the section of the ubiquity. The main problems were not due to the functioning of the system per se, but to the students’ internet connections (bandwidth, WIFI, etc.). The use of the hypervisor has enabled the management of computing and storage resources, making them available to the individual virtual machines. Access to the remote individual desktops from any location and device type has been possible.

The centralization of the applications facilitates their administration, configuration and updates, lowering their final licensing and installation costs. Virtual machines avoid software compatibility issues and new virtualization systems have new features that improve efficiency and cloud computing. Moreover, these engineering software require a large amount of resources (graphics memory, RAM, hard disk space, etc.), which not all students can afford. Thus, there is a preference for the virtualised environment over local installation on computers (3.89/5), although a considerable proportion of the total surveyed students have opted for the latter option. The computational performance of the environment passes with 3.04/5. As already mentioned, part of these deficiencies are due to poor connections of the students. It should be noted that, from a virtualization point of view, cores and physical memory do not impose a limit on virtual CPUs and total VM memory, since virtualization itself is responsible for distributing the physical CPUs in the virtual CPUs through time-sharing mechanisms.

The vast majority of students see the possibility of incorporating other new engineering software applications in the virtualised environment as very positive (4.30/5). The advantages of a system integrated with CAD/CAM/CAE tools are many: integration of design, calculation and manufacturing functions, shortening of the learning curve, elimination of file exchange incompatibilities, higher productivity and better control of engineering planning. If to all this is added the possibility of working with other types of engineering software from the same virtualised environment, it provides the student with an enormous potential for learning in the work environment. Furthermore, it has been demonstrated that virtualised computational environments are highly beneficial for carrying out PBL methods (Segrelles et al., 2017). The new characteristics of current students and future engineers, such as the millennial generation, require the implementation of a new learning rules and philosophy with a greater use of information and communication technology and the use of active learning strategies (Hernandez-de-Menendez & Morales-Menendez, 2019). This work shows a way of approaching this new paradigm in a virtual or hybrid teaching environment with dealing with the main themes of virtual distance: technology-enhanced distance engineering education, e-learning and virtual labs (Kocdar et al., 2020).

## Conclusions

The project developed and solved during a complete course has allowed the students learning by doing, and deal with many skills that are intended to be achieved in the master’s degree subjects involved. The project has been sequenced into concrete activities according to the most powerful phases of the product development process that go from the conceptual design of the product to the planning for its manufacture. However, the activities are not totally closed to allow the freedom, creativity and autonomous work of the student. The project raised is described in this work and has allowed connecting two different subjects transversally. The students showed a high satisfaction in the realization of the work. All this has been achieved through virtual teaching and work from home of students thanks to the virtualization system launched for this purpose in the University, which has been described in detail in this work. The operation of CAD/CAM/CAE tools, which usually requires significant computing resources, has worked well in this environment with very good results in the surveys conducted by students.

Definitively, all these points encourage the implementation of such projects in the university environment and to promote horizontal collaboration between related subjects to provide students with a more global and inclusive perspective. The proven usefulness of design and calculation tools with virtual machines also facilitates that they can be carried out from outside the classroom helping students to manage their time and the university.

## Abbreviations

ADME: Advanced Design in Mechanical Engineering (subject)
AME: Advanced Manufacturing Engineering (subject)
CAD: Computer-aided Design
CAE: Computer-aided Engineering
CAM: Computer-aided Manufacturing
CPU: central processing unit
CAx: Computer-aided technologies
PBL: Project-based Learning
VDI: Virtual Desktop Infrastructure
VM: Virtual Machine
vGPU: virtual Graphics Processing Unit
